# Establishing Priorities for the International Confederation of Plastic Surgery Societies

**DOI:** 10.1097/GOX.0000000000001878

**Published:** 2018-09-05

**Authors:** Rodney D. Cooter, Louise A. Brightman, Howard M. Clarke, Norma I. Cruz, Greg R. D. Evans, Kyung S. Koh, Robert X. Murphy, Graeme A. B. Perks, Hinne A. Rakhorst

**Affiliations:** From the * Department of Epidemiology and Preventive Medicine, Monash University, Melbourne, VIC, Australia; † Australasian Foundation for Plastic Surgery, St Leonards, NSW, Australia; ‡ International Confederation of Plastic Surgery Societies; § Hospital for Sick Children, University of Toronto, ON, Canada; ¶ Division of Plastic Surgery, University of Puerto Rico, San Juan, Puerto Rico; ‖ Humanitarian Committee, ICOPLAST; ** Department of Plastic Surgery, University of California, Oakland, Calif.; †† Asan Medical Centre, University of Ulsan, Seoul, Korea; ‡‡ Lehigh Valley Health Network, Allentown, Pa.; §§ Morsani College of Medicine, University of South Florida, Tampa, Fla.; ¶¶ Breast and Cosmetic Implant Registry (BCIR), United Kingdom; ‖‖ Department of Plastic, Reconstructive and Burns Surgery, Nottingham University Hospitals NHS Trust, Nottingham, United Kingdom; *** Dutch Breast Implant Registry (DBIR), The Netherlands; ††† Department of Plastic, Reconstructive and Hand Surgery, Medisch Spectrum Twente, Enschede, The Netherlands.

## Abstract

**Background:**

The mission of the International Confederation of Plastic Surgery Societies (ICOPLAST) is to improve patient outcomes through collaboratively structured processes in education, advocacy and communication. This article explains how we approached the task of establishing priorities for this nascent confederation in an equitable and achievable manner.

**Methods:**

In late 2016, an online survey was sent to the inaugural 62 ICOPLAST member national societies for dissemination to their respective plastic surgeon members. Functional domains and proposed initiatives were ranked according to their level of importance by individual plastic surgeons.

**Results:**

The survey was completed by 572 plastic surgeons. As a functional domain, education was highly ranked by 75.3% of respondents, followed by patient safety (67.4%), communication (59.3%), humanitarian (46.6%), regulation (41.2%), and advocacy (41.1%). Respondents also ranked individual initiatives within each domain to produce a compilation list of the top 13 initiatives of importance.

**Conclusion:**

This study has identified priorities of importance to ICOPLAST members, which will aid in building a strategic framework and enhancing outcomes for patients, plastic surgeons, and the field of plastic surgery more broadly.

## Abstract 2

Supplemental Digital Content is available in the text.

## INTRODUCTION

The International Confederation of Plastic Surgery Societies (ICOPLAST) was founded in 2016 with the global goals of improving patient safety and outcomes in plastic surgery and enhancing the quality of aesthetic and reconstructive surgery through education, communication, and advocacy. These goals align with the core values of ICOPLAST: to provide benefits for patients, plastic surgeons, and the field of plastic surgery more broadly.^1,2^

ICOPLAST is a confederation of national plastic surgery societies. Geographically, ICOPLAST membership spans 5 continents, more than 60 countries, and represents over 20,000 plastic surgeons (Table 1). Each national society is represented on the Council of National Delegates, which is ICOPLAST’s governing body. In addition, regional representatives are democratically elected to form the Board of Directors who oversee the management of ICOPLAST and who are accountable to the Council of National Delegates. Although there are no individual members of ICOPLAST, every plastic surgeon has a voice by virtue of being a member of a national plastic surgery society. ICOPLAST membership is designed to be a union of common purpose that does not impact upon the independence of any national society.

**Table 1.**
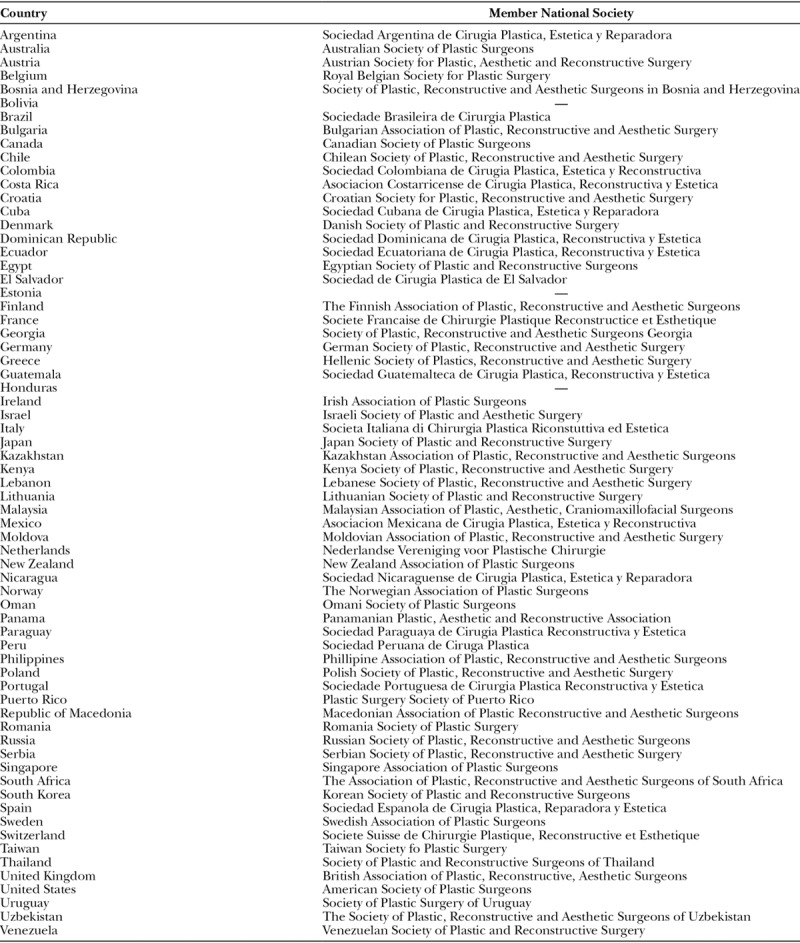
ICOPLAST Member Countries and National Plastic Surgery Societies

To develop a new international plastic surgical society from scratch is a formidable challenge. In a concerted effort to embrace all national plastic surgical societies in an equitable manner, this survey was constructed to provide a snapshot of the ICOPLAST membership demographic and to identify the needs, visions, and ambitions of plastic surgeons globally. The ultimate aim of this survey was to establish a set of priorities to assist in developing a balanced strategic plan for ICOPLAST activities.

## METHODS

### Study Design

Before their distribution as a survey, a battery of questions was reviewed by ICOPLAST Directors. The final survey comprised 33 questions, some of which had prespecified fields and others open response options for clarification of demographic information and feedback. Questions were categorized into: Respondent demographics; Practice, Academic, and Research characteristics; and specific inquiry into the domains of Education; Patient Safety; Communication; Advocacy; Humanitarian; and Regulation. The survey included a question regarding the geographical region of plastic surgery practice but not the specific country of origin or country of practice. The majority of the questions employed a 5-point Likert Scale to obtain the level of value placed on each item. The Likert Scale ranged from “no value” to “high value.” It was designed to ensure sufficient capture of demographic information and communication methods and covering a wide range of topics relevant to plastic surgeons and contemporary plastic surgical practice. The full survey can be found in **Supplemental Digital Content 1**, http://links.lww.com/PRSGO/A818.

The survey was written in English and sent to ICOPLAST Directors with an invitation to facilitate translation into their respective regional languages. Most Directors agreed that English was the dominant professional language for plastic surgeons internationally; therefore, the final survey was distributed in English. The survey was initially sent via e-mail in August 2016. Three reminder e-mails were sent before the survey closing in January 2017. Although the authors were mindful of the principles outlined in the Declaration of Helsinki, formal Human Research and Ethics approval was not required as the project did not involve patient care or utilize clinical data, and the risk to participants was deemed to be negligible in accordance with the National Health and Medical Research Council National Statement on Ethical Conduct in Human Research.^3^

### Study Participants

Surveys were sent to the 62-member national societies for dissemination to individual members of their societies. As ICOPLAST was still in an early stage of establishment, contact with all member countries’ plastic surgeons was not feasible in the timeframe available.

### Data Collection and Analysis

Survey responses were entered into an Excel spread sheet in a deidentified manner. For those questions that employed the Likert Scale, the 2 highest rankings (very good and highly valuable) were combined and then divided by the respective response count. This pooled the 2 highest rankings for each question into a single percentage format and allowed the top 2 participant preferences to be considered equally.

Analysis of the findings and subsequent planning was performed in conjunction with those co-authors who also hold the position of ICOPLAST Director. Strategies to accomplish the ranked priorities were allotted timeframes based on ICOPLAST resources.

## RESULTS

A total of 572 responses were received. The secondary dissemination of the survey from national society delegates to individual surgeons prevents the ability to calculate an accurate response rate; however, the percentage capture of membership countries by regions is outlined in Table 2. Respondent demographics are presented in Table 2. Nonresponse rates for individual items within Table 2 varied from 0.9% to 5.4%. The majority of respondents were males (78.4%) aged 30–70 years from Europe (41.3%), North Asia (25.5%), and North America (19.2%). Nonboard members and nonnational delegates of the member countries were the predominant responders (84.0%). Connectivity is also outlined in Table 2.

**Table 2.**
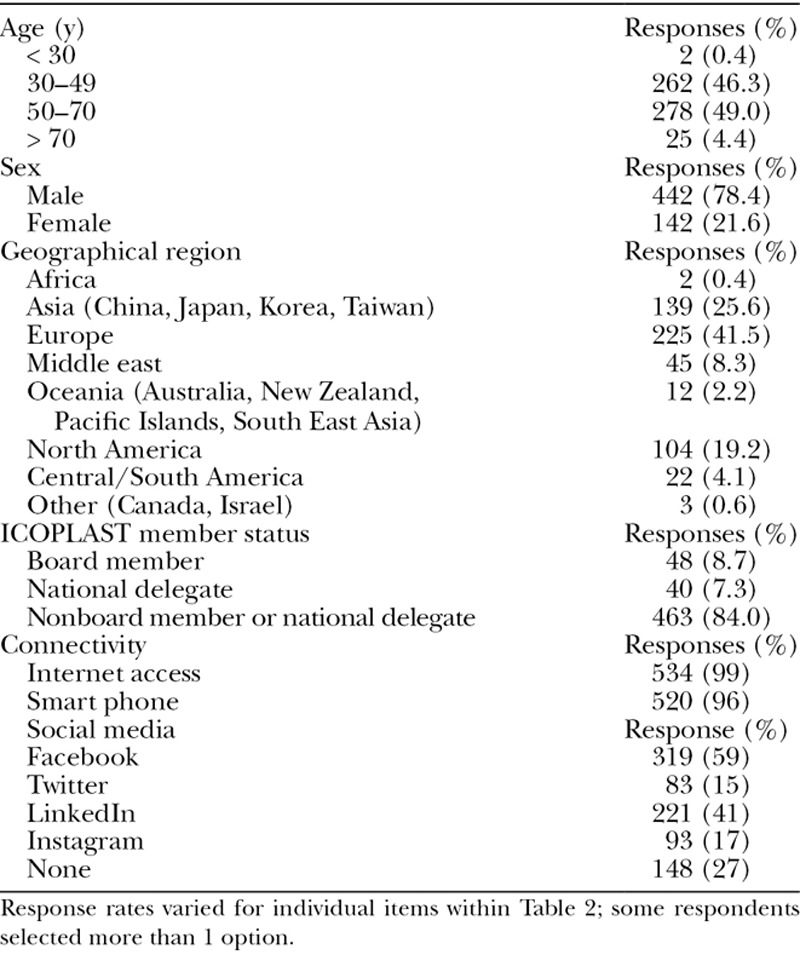
Respondent Demographics

Practice characteristics, additional academic qualifications, and research activities of respondents are outlined in Table 3. Nonresponse rates for individual items within Table 3 varied from 1.9% to 5.9%. Private practice was the most common form of current occupation (38.1%) with solo private practitioners almost double the number of group private practices (32.9% and 16.9%, respectively). Mixed reconstructive and aesthetic practice was the most common type of plastic surgical endeavor (44.9%) followed by predominantly reconstructive (26.6%) and aesthetic (21.6%). Of the 3.0% of respondents who selected “other” as their type of practice, the vast majority reported hand surgery.

**Table 3.**
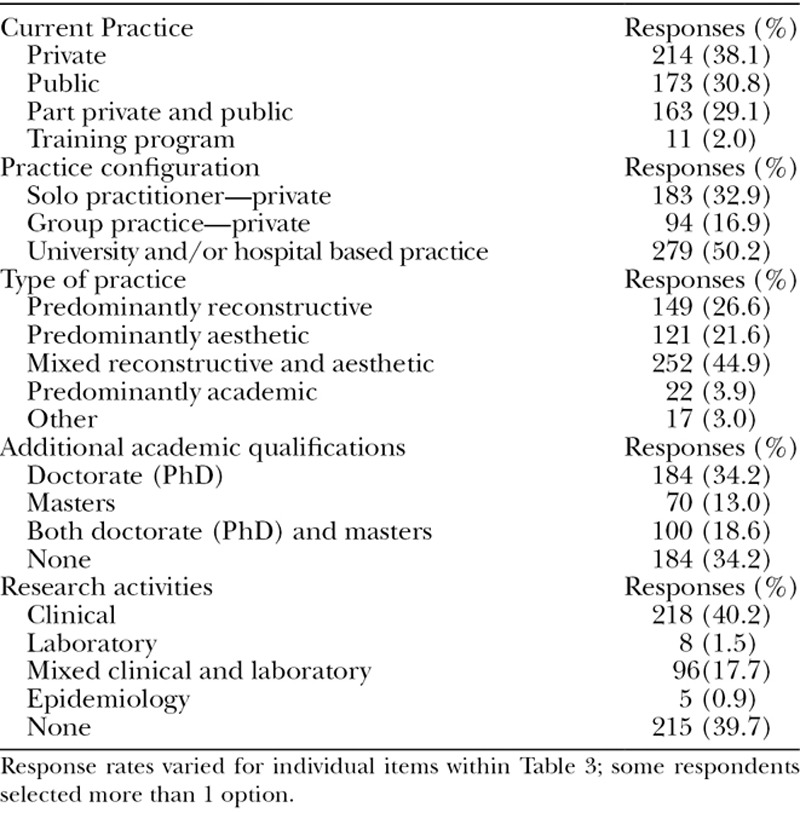
Practice, Academic, and Research Characteristics

### Domains of Value for ICOPLAST Activities and Functions

**Fig. 1.**
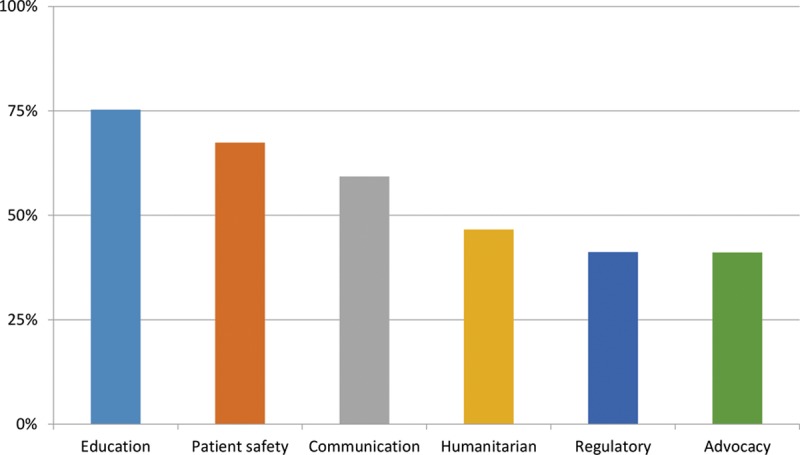
Domains of value for ICOPLAST activities and functions.

Respondents ranked the value they place on proposed ICOPLAST activities and functions. This resulted in the identification of 6 broad domains (Fig. 1). As a valuable functional domain, education was selected by 75.3% of respondents, followed by patient safety (67.4%) and communication (59.3%). Lower ranked domains of value included humanitarian (46.6%), regulation (41.2%), and advocacy (41.1%). Within each domain, respondents ranked several individual initiatives, which resulted in a compilation list of the top 13 ranked initiatives (Table 4).

**Table 4.**
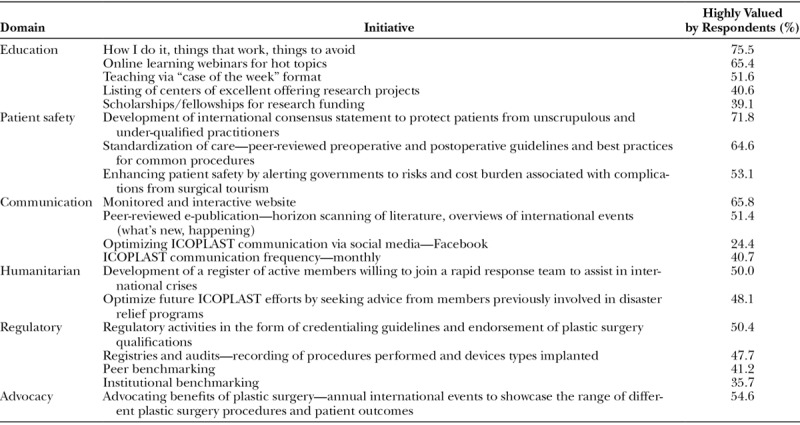
Top 13 Ranked Initiatives within the 5 Most Valued Domains

### Top Ranked Initiatives

Practical education of plastic surgery techniques using webinar delivery was considered the highest priority by respondents. Specific initiatives of interest included advice on “How I do it” and “Things that work” (75.7%) and “Hot topics” (65.4%). Patient safety initiatives supported by international consensus statements were considered a priority by 71.8%. Standardization of care by generating peer-reviewed guidelines of international significance features strongly at 64.6%. Additionally, enhancing patient safety by alerting governments to the cost burden of complications associated with surgical tourism was also ranked highly at 53.1% (Table 4).

An interactive ICOPLAST website (65.8%) was ranked strongly by respondents, as was the concept of reviewing existing plastic surgery articles via a peer-reviewed ICOPLAST e-publication (51.4%). International efforts to advocate the benefits of plastic surgery and humanitarian endeavors were initiatives of high importance to 54.6% and 50.0%, respectively. Credentialing guidelines and endorsement of plastic surgeon qualifications (50.4%) and data management strategies such as audits and registries to improve patient outcomes (47.7%) were also considered priorities of importance and ranked accordingly (Table 4).

## DISCUSSION

The demographic profile of respondents highlights the need for ICOPLAST to be a responsive organization and sensitive to a broad spectrum of ages and cultures. Further, the high value placed on the abovementioned initiatives reflects the need for ICOPLAST to plan a comprehensive portfolio of professional deliverables in several domains.

Although there are several limitations including an unknown response rate (due to the secondary dissemination of the survey by national ICOPLAST delegates) and the potential for selection bias based on the distribution method, the diverse demographic data provide some reassurance that we have sourced representative information from ICOPLAST member societies. Although there was an unequal spread of respondents geographically, the fact that all major regions were represented to a varying degree suggests that the findings can be generalized to the growing ICOPLAST community.

On the basis of the survey results, strategic planning was assigned in an effort to accomplish the priorities identified within the top 13 ranked initiatives. An implementation strategy was formulated via consensus by those co-authors who also hold the position of ICOPLAST Director. Timeframes of immediate (2017 to mid-2018), short-term (2018), and longer term (2019–2020) were allotted based on ICOPLAST resources.

### Immediate Priorities (2017 to mid-2018)

#### Funding

Funding was the highest priority to enable the committees to accomplish their assigned tasks. It was determined that each member country would be required to contribute according to the number of active plastic surgeons within their country’s national society. An annual fee of 10 Euros per active national society member was the standard level of dues; however, a reduced fee was made available to those countries recognized as low income.

#### Committees

Five committees have been established; these include a Website Development Committee, a Patient Safety Committee, an Education Committee, a Humanitarian Committee, and a Professional Standards Committee. Each committee has been populated with a balanced group of members from each of the major regions including representation from North America, South America, Middle East, Europe, Asia, and Oceania.

### Short-term Priorities (2018)

#### Website Development

Fundamental to the interconnectedness of ICOPLAST is an effective website with which to interact, to deliver online learning topics, and to store the content of webinars for future access by members. This will enable a monitored and interactive website, online learning webinars for hot topics and the provision of teaching using a “Case of the week” format (initiatives 3, 4, and 8, respectively). The website will also be the future portal for standardizing pre- and postoperative guidelines and best practices for common procedures (initiative 5) and peer-reviewed ICOPLAST e-publication (initiative 9).

#### Patient Safety Projects

Patient safety was considered one of the highest priorities as evidenced by the interest in development of an international consensus statement to protect patients from unscrupulous and under-qualified practitioners (initiative 2). Standardization of care through peer-reviewed pre- and postoperative guidelines and best practices for common procedures (initiative 5) and enhancing patient safety by alerting governments to the risks and cost burden associated with surgical tourism complications (initiative 7) also featured. To that end, a project has been commenced to explore the process of Informed Consent, with a particular emphasis on surgical tourism where evidence is mounting that the consenting processes would fall short of that recommended by most national plastic surgical societies.^4–6^

By using a collaborative approach from member countries, ICOPLAST is uniquely placed to generate an international best practice informed consent guide for dissemination globally in an effort to address initiatives 5 and 7. In response to the latest international consensus statement on Breast Implant Associated Anaplastic Large Cell Lymphoma, the Patient Safety Committee has disseminated practical guidelines for members, which is consistent with initiative 5. ICOPLAST will also look to build on the recent work by Brightman et al.^7^ entitled “Cosmetic tourism for breast augmentation: a systematic review,” which highlighted themes consistent with initiative 7.

#### Educational Offerings

Within the remit of the Educational Committee will be techniques that work, “How I do it” and “Things to avoid” (initiative 1). Although ICOPLAST is not planning to hold stand-alone conferences, it is planning short symposia on specific topics to be held in conjunction with the national meeting of a member society. This model was trialed at the 2017 Australian Society of Plastic Surgeons where the ICOPLAST Directors presented their chosen educational topic. In a more targeted fashion, a symposium on recent advances in plastic surgery of the breast was combined with the meeting of the Egyptian Society of Plastic and Reconstructive Surgeons Egyptian Society in Luxor, March 2018.

### Longer Term Priorities (2019–2020)

#### Public Education and Plastic Surgery Advocacy

An annual event showcasing the positivity of plastic and reconstructive surgery in the lives of everyday citizens was ranked favorably (initiative 6). To address this initiative, ICOPLAST has formed a “Wake Up to Plastic Surgery Campaign Task Force” to profile unique stories and digital content that showcases the level of impact and innovation from plastic surgery globally. This is being planned as an annual campaign to be themed to increase the public’s awareness of the wide range of plastic surgery involvement in the wider community. The “Wake Up to Plastic Surgery” theme for 2018 is “Prevent the Bite” to promote better awareness of the impact of animal bites on patients world-wide. Plastic surgeons reconstruct 10s of 1000s of patients annually after animal bites so this campaign will inform the public with simple key messages about the impact of the plastic surgery specialty.

#### Humanitarian Planning

ICOPLAST in its efforts to set standards and promote the work of plastic surgeons and related charitable care organizations providing volunteer services to areas of need has published a position paper “Best practices and standards for humanitarian initiatives” (available at www.icoplast.org). The Humanitarian Committee will now start to collaborate with the World Health Organization on a common document regarding standards for humanitarian initiatives. In addition, a Visiting Humanitarian Professorship has been created. On an annual basis a professor, upon request from a local plastic surgery society, will teach skills and empower plastic surgeons and local health care professionals to provide care to their communities.

Among other activities the Humanitarian Committee will be addressing the development of a register of active members willing to join a rapid response team to assist in the event of international crises (initiative 11) and optimizing future ICOPLAST efforts by seeking advice from members previously involved in disaster relief programs (initiative 12).

#### Professional Standards

The development of credentialing guidelines for the endorsement of plastic surgery qualifications (initiative 10) will fall within the scope of the Professional Standards Committee and will involve the longer term objective of defining “What is a plastic surgeon?”. The purpose of this initiative is to educate the public rather than ICOPLAST becoming an arbiter of determining qualifications, which will continue to remain within the scope of existing professional organizations.

#### Registries and Audit

Registries and audits for the purpose of recording procedures performed and device types implanted (initiative 13) will aim to assist current efforts^8,9^ to monitor implantable device performance at an international level, especially, in the first instance, breast implants for both reconstructive and aesthetic purposes. Audits will ultimately facilitate international benchmarking of plastic surgical procedure outcomes.

## CONCLUSIONS

This study has helped to identify overarching domains and individual initiatives of importance to ICOPLAST members. This, in turn, has provided the basis for a strategic framework upon which ICOPLAST can work toward delivering outcomes to benefit patients, plastic surgeons and the field of plastic surgery more broadly. Priorities have been set and goals have been outlined according to realistic timeframes and ICOPLAST resources. Immediate priorities include funding of key committees for website development, patient safety, education, humanitarian endeavors and professional standards. Short-term priorities will expand on the delivery an interactive website, the provision of education to ICOPLAST members and commencement on several patient safety projects. Longer term priorities will focus on delivering public education, plastic surgery advocacy, humanitarian planning, professional standards, and data management strategies.

ICOPLAST is an evolving confederation that welcomes global participation and collaboration. Those who would like to be involved are encouraged to contact us on info@icoplast.org or e-mail/call your regional Board Member.^1^

## ACKNOWLEDGMENTS

The authors acknowledge Carol Lazier and Jacqueline Luna for their executive assistance with the survey, along with Lucie Lessard, Sean Carrol, and Andrzej Piatkowski for their ongoing efforts to help ICOPLAST implement the initiatives delineated in this article. The authors also acknowledge Hassan Badran, Julio Daniel Kirschbaum, and Takashi Nakatsuka. Finally, the authors are grateful to Ingrid Hopper for her assistance with drafting and editing the article.

## Supplementary Material
