# Supplementary material for: Establishing Priorities for the International Confederation of Plastic Surgery Societies
Source: Plast Reconstr Surg Glob Open. 2018 Sep 5;6(9):e1878. doi: 10.1097/GOX.0000000000001878 (PMC6191229; doi:10.1097/GOX.0000000000001878)
Supplement: Supplementary file 1 [file gox-6-e1878-s001.pdf]

ICOPLAST

INTERNATIONAL CONFEDERATION OF PLASTIC SURGERY SOCIETIES

ICOPLAST

Introduction

ICOPLAST is the newly formed International Confederation of Plastic Surgery Societies

This survey presents a unique opportunity: it is YOUR chance to have YOUR say about OUR new organization, ICOPLAST, to help shape the future for ALL plastic surgeons and their PATIENTS globally.

Aims:

To inform/educate about ICOPLAST

To get feedback about priorities of issues to address in short-term.

To build a longer-term vision for future activities.

ICOPLAST

INTERNATIONAL CONFEDERATION OF PLASTIC SURGERY SOCIETIES

ICOPLAST

Demographic Information

1. In which age bracket will you be on 1 January 2017?

- ◯ < 30
- ◯ 30 - 49
- ◯ 40 - 49
- ◯ 50 - 59
- ◯ 60 - 70
- ◯ >70

2. What type of member of ICOPLAST are you?

- ◯ ICOPLAST Board Member
- ◯ ICOPLAST National Delegate *(Not an ICOPLAST Board Member)*
- ◯ Active Member of National Society *(Not an ICOPLAST Board Member or National Delegate)*

3. Are you male or female?

- ◯ Male
- ◯ Female

4. Current Practice:

- ◯ Private Practice
- ◯ Public Practice
- ◯ Part Private/Part Public
- ◯ In Training Program

5. Practice Configuration

- ◯ Sole Practitioner - private
- ◯ Group Practice – private
- ◯ University/Hospital – based practice

6. Type of Practice

- ◯ Predominantly Reconstructive
- ◯ Predominantly Aesthetic
- ◯ Mixed Reconstructive & Aesthetic
- ◯ Predominantly Academic
- ◯ Other (please specify)

7. Approximately, what is the amount of the individual member dues paid to the national society you belong to?

ICOPLAST

Qualifications/Research

8. In addition to your plastic surgery qualifications, do you hold an academic degree? (Check all that apply.)

- ○ Doctorate
- ○ Masters
- ○ Both Doctorate and Masters
- ○ No

9. Are you actively involved in research?

- ○ Clinical
- ○ Laboratory
- ○ Mixed clinical/laboratory
- ○ Epidemiology
- ○ No

10. In which geographic region do you practice? (Check all that apply.)

- [ ] Africa
- [ ] Asia (China, Japan, Korea, Taiwan)
- [ ] Europe (EC countries)
- [ ] Middle East
- [ ] Oceania (Australia, NZ, Pacific Islands, SE Asia)
- [ ] North America
- [ ] Central/South America
- [ ] Other (please specify)

11. How much longer do you intend to practice after 1 January, 2017?

- ◯ < 5 years
- ◯ 5-10 years
- ◯ 11-20 years
- ◯ 21-30 years
- ◯ > 30 years

12. Do you have internet access?

- ◯ Yes, always
- ◯ No, never
- ◯ Sometimes
- ◯ Will have within five years

13. Do you possess a smart phone?

- ◯ Yes
- ◯ No
- ◯ Expect to have one in three years

14. What social media channels do you use? (Check all that apply)

- [ ] Facebook
- [ ] Twitter
- [ ] LinkedIn
- [ ] Instagram
- [ ] None
- [ ] Other (please specify)

15. Of which plastic surgery-related societies are you a member? (Check all that apply.)

- [ ] National Plastic & Reconstructive Surgery Society
- [ ] Regional Aesthetic Surgery Society
- [ ] ISAPS - International Society of Aesthetic Plastic Surgery
- [ ] ESPRAS - European Society of Plastic, Reconstructive and Aesthetic Surgery
- [ ] FILACP - Federación Ibero Latino Americana de Cirurgía Plàstica
- [ ] AAPS - American Association of Plastic Surgeons
- [ ] EURAPS - European Association of Plastic Surgeons
- [ ] ISPRES - International Society of Plastic and Regenerative Surgeons
- [ ] World Society for Reconstructive Microsurgery
- [ ] ICPF - International Cleft Lip and Palate Foundation
- [ ] IFSSH - International Federation of Societies for Surgery of the Hand
- [ ] Other (please specify)

16. Other specialty societies that I am a member of relating to the following areas?

- [ ] Bariatric
- [ ] Breast
- [ ] Burns
- [ ] Clefts
- [ ] Cosmetic
- [ ] Craniofacial
- [ ] Data Management
- [ ] Fat Transfer
- [ ] Gender Reassignment
- [ ] Hand
- [ ] Head & Neck
- [ ] History
- [ ] Laser
- [ ] Maxillofacial
- [ ] Melanoma
- [ ] Microsurgical
- [ ] Nerve
- [ ] Non-Surgical Treatments
- [ ] Oculoplastic
- [ ] Research
- [ ] Tissue Engineering
- [ ] Transplant
- [ ] Wound
- [ ] Other (please specify)

INTERNATIONAL CONFEDERATION OF PLASTIC SURGERY SOCIETIES

ICOPLAST

17. Please rank the items below in the order of its value to you in a medical association/society.

	No Value	Maybe	Good	Very Good	High Value
Education	◯	◯	◯	◯	◯
Patient Safety	◯	◯	◯	◯	◯
Communication	◯	◯	◯	◯	◯
Regulatory	◯	◯	◯	◯	◯
Advocacy	◯	◯	◯	◯	◯
Humanitarian	◯	◯	◯	◯	◯

Other (please specify)

ICOPLAST

INTERNATIONAL CONFEDERATION OF PLASTIC SURGERY SOCIETIES

ICOPLAST

Education

To assist you to improve outcomes for your patients, please rank each of the following initiatives that ICOPLAST might offer

18. Teaching

	No Value	Maybe	Good	Very Good	High Value
On-line learning: Webinars on "hot" topics	◯	◯	◯	◯	◯
Diagnostic/management: Case-of-the-week	◯	◯	◯	◯	◯
Techniques: How-I-do it; things that work, things to avoid	◯	◯	◯	◯	◯
Medicolegal: Avoiding legal traps	◯	◯	◯	◯	◯
CME: Annual personal audit of on-line involvement	◯	◯	◯	◯	◯
Clinical Fellowships: Job Vacancies	◯	◯	◯	◯	◯

19. Publishing

	No Value	Maybe	Good	Very Good	High Value
What's New: Horizon scanning of existing publications	○	○	○	○	○
What's Happening: Overview of international educational events	○	○	○	○	○
An e-publication: An on-line ICOPLAST peer-reviewed publication	○	○	○	○	○

Other (please specify)

20. Research

	No Value	Maybe	Good	Very Good	High Value
Compilation of a list of centers of excellence offering research projects	○	○	○	○	○
Compilation of a list of scholarships/fellowships available to fund research	○	○	○	○	○

ICOPLAST

INTERNATIONAL CONFEDERATION OF PLASTIC SURGERY SOCIETIES

ICOPLAST

Patient Safety

Please rank the following potential activities that may be offered from ICOPLAST

21. Standardizing

	No Value	Maybe	Good	Very Good	High Value
Peer-reviewed **pre-operative** guidelines/best practices for common procedures	◯	◯	◯	◯	◯
Peer-reviewed **post-operative** guidelines/best practices for common procedures	◯	◯	◯	◯	◯

22. Ethical Practices

	No Value	Maybe	Good	Very Good	High Value
For advertising	◯	◯	◯	◯	◯
For your practice	◯	◯	◯	◯	◯

23. Registries & Audit

	No Value	Maybe	Good	Very Good	High Value
For devices inserted	○	○	○	○	○
For operations performed	○	○	○	○	○
Benchmarking outcomes against peers	○	○	○	○	○
Institutional benchmarking	○	○	○	○	○

ICOPLAST
INTERNATIONAL CONFEDERATION OF PLASTIC SURGERY SOCIETIES

ICOPLAST

Communication

Please let us know how ICOPLAST can optimize our communication with all plastic surgeons globally by ranking the options below:

24. Internet

	No Value	Maybe	Good	Very Good	High Value
Monitored & Interactive Website	◯	◯	◯	◯	◯
Facebook	◯	◯	◯	◯	◯
Twitter	◯	◯	◯	◯	◯
LinkedIn	◯	◯	◯	◯	◯
Instagram	◯	◯	◯	◯	◯

Other (please specify)

25. Frequency of email blasts of events/notices/employment opportunities

	No Value	Maybe	Good	Very Good	High Value
Weekly	◯	◯	◯	◯	◯
Every Two Weeks	◯	◯	◯	◯	◯
Monthly	◯	◯	◯	◯	◯

ICOPLAST
INTERNATIONAL CONFEDERATION
OF PLASTIC SURGERY SOCIETIES

ICOPLAST

Regulatory

As all plastic surgeons work within regulated environments, the opportunity exists for ICOPLAST member societies to assist each other to achieve better outcomes universally. Please rate the relevance to your society or practice of the following potential initiatives:

26. Accreditation Guidelines

	No Value	Maybe	Good	Very Good	High Value
For office-based (local anesthesia) procedures	◯	◯	◯	◯	◯
For ambulatory day cases	◯	◯	◯	◯	◯
For general anesthesia cases	◯	◯	◯	◯	◯

Other (please specify)

27. Credentialing Guidelines

	No Value	Maybe	Good	Very Good	High Value
Endorsement of plastic surgery qualifications	◯	◯	◯	◯	◯
Endorsement of minimum caseload per annum	◯	◯	◯	◯	◯

ICOPLAST

INTERNATIONAL CONFEDERATION OF PLASTIC SURGERY SOCIETIES

ICOPLAST

Advocacy

It is apparent that each society has similar challenges with their relationships with governing bodies. The possibility exists that ICOPLAST could harness the strengths of some mature advocacy programs to help support sister societies with advocacy programs in earlier stages of evolution.

Please rate the examples provided below:

28. Surgical Tourism

	No Value	Maybe	Good	Very Good	High Value
Develop an "international voice" to enhance patient safety by alerting governments to the cost burden (of complications) and risks	○	○	○	○	○

29. Unregulated Plastic Surgery

	No Value	Maybe	Good	Very Good	High Value
Develop a consensus statement internationally to protect patients from unscrupulous, under-qualified practitioners	○	○	○	○	○

30. Showcase ICOPLAST and its logo with a "Wakeup to Plastic Surgery" International Campaign

	No Value	Maybe	Good	Very Good	High Value
Develop a new "New Day Dawning" style event at the same time on a particular day each year to showcase ***different types*** and the positivity of plastic & reconstructive surgery to the lives of everyday citizens around the world each year.	○	○	○	○	○

ICOPLAST
INTERNATIONAL CONFEDERATION OF PLASTIC SURGERY SOCIETIES

ICOPLAST

Humanitarian

It is well recognized that many plastic surgeons are involved in humanitarian programs internationally. ICOPLAST's vision is to support these endeavors, not to duplicate them. Support may come in style of the following initiatives.

31. Develop a Register of Existing Humanitarian Programs

	No Value	Maybe	Good	Very Good	High Value
Develop a register of active members willing to join a 'rapid response' team to assist in an international crisis	○	○	○	○	○
Seek advice from members involved in previous disaster relief programs to ensure any ICOPLAST effort is optimized	○	○	○	○	○

32. What programs are you involved with?

33. Do you have any other thoughts or suggestions as to what you would like to see ICOPLAST offer or participate in?

ICOPLAST

INTERNATIONAL CONFEDERATION
OF PLASTIC SURGERY SOCIETIES

ICOPLAST

Thank you for your participation!

We look forward to working together with you.
